# Supplementary material for: Brain Health and Cognition in Older Adults: Roadmap and Milestones towards the Implementation of Preventive Strategies
Source: Brain Sci. 2024 Jan 6;14(1):55. doi: 10.3390/brainsci14010055 (PMC10813413; doi:10.3390/brainsci14010055)
Supplement: Supplementary file 1 [file brainsci-14-00055-s001.zip › brainsci-2770342-supplementary.pdf]

Monza BHS Template

Federico Emanuele Pozzi, Giulia Remoli, Lucio Tremolizzo, Ildebrando Appollonio, Carlo Ferrarese, Luca Cuffaro

Reason for access
Scegliere un elemento.

History:
n -year old *Man/woman*. Married/widow. Lives with Fare clic o toccare qui per immettere il testo..
Since *n* years complains Fare clic o toccare qui per immettere il testo. *Examples:*
Memory: Fare clic o toccare qui per immettere il testo. The memory deficits have improved/worsened compared to the previous year. Compared to peer, memory is subjectively worse/better.
Focus: Fare clic o toccare qui per immettere il testo.
Orientation in space: Fare clic o toccare qui per immettere il testo.
Speech and language (and bilinguism): Fare clic o toccare qui per immettere il testo.
Daily life activities: *including device use and finance management*. ADL *n* IADL *n*
Mood: Fare clic o toccare qui per immettere il testo.
Social habits: Fare clic o toccare qui per immettere il testo.
Autonomic disturbances: *urination, stipsis, nocturia, orthostatic hypotension, excessive sweating*
Hallucinations: *visual, auditory, sense of presence*
Sleep: *Vivid dreams/RBD. Insomnia. Sonnolenza diurna. OSAS.*
Falls: Scegliere un elemento.
Hobbies: Fare clic o toccare qui per immettere il testo.
Diet: Scegliere un elemento.
Physical activity: Scegliere un elemento.
Further considerations: Fare clic o toccare qui per immettere il testo.
Family history: Scegliere un elemento.
Education: *years. Occupation/last occupation*.

Comorbidities: *diabetes? hypertension? TBI? Obesity? Depression? Hearing issues? A-fib? Visual issues?*
Drugs: Fare clic o toccare qui per immettere il testo.
Smoke, weekly alcohol units:

packs	years	pack-years	0	
beer (33 cl)	wine (glasses)	spirits (glasses)		Units 0

*select the cell to be calculated, right-click and select "update field"

Diagnostic tests:

- Brain MRI/CT *year*: MTA: sx *value* dx *value*, Fazekas: *value*, Lacune: *n*, ARWMC: *value*, GCA: *n*, PCA: sx *value* dx *value*, CMB: *n* of which lobar *n*
- Brain PET-FDG *year*: Fare clic o toccare qui per immettere il testo.
- NPS tests *year*: Fare clic o toccare qui per immettere il testo.
- Lumbal puncture *year*: (A+ T+ N+)

Aβ42		Aβ40		p-tau	t-tau
Aβ42/40	**!Divisione per zero**	Aβ42/p-tau	**!Divisione per zero**		

- Amyloid-PET *year*: Scegliere un elemento.
- *Other*
- ApoE: Scegliere un elemento.

Clinical examination:
Neurological examination: Fare clic o toccare qui per immettere il testo.
MMSE: *n*/30; recall *n*/3 (*n*/3 after *phonetic/semantic* cue)
MoCA: *n* /30

FAB: *n* /18
Praxis: Fare clic o toccare qui per immettere il testo.
Recent news facts: Fare clic o toccare qui per immettere il testo.
Blood pressure (clinostatic/orthostatic): Fare clic o toccare qui per immettere il testo.
Hyperlipemia: Fare clic o toccare qui per immettere il testo.
Weight (kg) Height (m) BMI **!Divisione per zero**
*select the cell to be calculated, right-click and select “update field”

Risk:
CAIDE (39-64): *insert the risk calculated with the app*
https://brainhealthservice.shinyapps.io/Riskcalculator/
ADappt (45-85): Fare clic o toccare qui per immettere il testo.
https://adappt.health/en/tool/
BDSI (65-79): Fare clic o toccare qui per immettere il testo.
https://campuslifeservices.ucsf.edu/clsforms/documentsmedia/dementiarisk/
Interest in trials: Fare clic o toccare qui per immettere il testo.
Informed consent for study signed Fare clic o toccare qui per immettere il testo.

Conclusions:
SCD/MCI

SCD plus criteria:
☐ Cognitive decline confirmed by informant
☐ Complaints since < 5 years
☐ Performance worse than peers
☐ Isolated amnesic cognitive decline
☐ Age > 60 years
☐ APOE4+

If functional: https://neurosymptoms.org/en/symptoms/fnd-symptoms/functional-cognitive-symptoms/
Work-up and recommendations:
- **Brain MRI** Fare clic o toccare qui per immettere il testo.
- Brain amyloid-PET/PET-FDG (if high risk) Fare clic o toccare qui per immettere il testo.
- Lumbar puncture (if high risk) Fare clic o toccare qui per immettere il testo.
- **NPS tests** Fare clic o toccare qui per immettere il testo.
- **Blood tests for biobanking / plasma biomarkers** Fare clic o toccare qui per immettere il testo.
- Polysomnography Scegliere un elemento.
- Blood pressure holter: Fare clic o toccare qui per immettere il testo.
- ApoE (if high risk): Fare clic o toccare qui per immettere il testo.
- Diet: Fare clic o toccare qui per immettere il testo.
- Physical activity: Fare clic o toccare qui per immettere il testo.
- Cognitive stimulation: Fare clic o toccare qui per immettere il testo.
- Speech therapy: Fare clic o toccare qui per immettere il testo.
- ENT referral: Fare clic o toccare qui per immettere il testo.
- Ophtalmologist referral: Fare clic o toccare qui per immettere il testo.
- Psychiatric referral: Fare clic o toccare qui per immettere il testo.
- *Evaluation at 1 year at BHS/Memory clinic referral*

Email

Doctor signature
